# Oxidative Stress to the Cornea, Changes in Corneal Optical Properties, and Advances in Treatment of Corneal Oxidative Injuries

**DOI:** 10.1155/2015/591530

**Published:** 2015-03-11

**Authors:** Cestmir Cejka, Jitka Cejkova

**Affiliations:** Laboratory of Eye Histochemistry and Pharmacology, Department of Neuroscience, Institute of Experimental Medicine, Academy of Sciences of the Czech Republic, Vídeňská 1083, 14220 Prague 4, Czech Republic

## Abstract

Oxidative stress is involved in many ocular diseases and injuries. The imbalance between oxidants and antioxidants in favour of oxidants (oxidative stress) leads to the damage and may be highly involved in ocular aging processes. The anterior eye segment and mainly the cornea are directly exposed to noxae of external environment, such as air pollution, radiation, cigarette smoke, vapors or gases from household cleaning products, chemical burns from splashes of industrial chemicals, and danger from potential oxidative damage evoked by them. Oxidative stress may initiate or develop ocular injury resulting in decreased visual acuity or even vision loss. The role of oxidative stress in the pathogenesis of ocular diseases with particular attention to oxidative stress in the cornea and changes in corneal optical properties are discussed. Advances in the treatment of corneal oxidative injuries or diseases are shown.

## 1. Introduction

An imbalance between reactive oxygen species (ROS) generation and the capacity of antioxidant ROS scavenging systems results in oxidative stress associated with cell damage, such as lipid peroxidation of membranes, oxidative changes in proteins, and oxidative damage to DNA [1]. In humans, oxidative stress is involved in many eye diseases, including cataract, uveitis, retinopathy of prematurity, age-related macular degeneration, and primary open-angle glaucoma [2–11]. Spector [2] summarized data from cataract patients and described significantly elevated levels of hydrogenium peroxide in their lenses and aqueous humor. Hydrogenium peroxide is the major oxidant involved in lens opacification and cataract formation. During uveitis, inflammation has a strong correlation with the oxidative stress [3]. In the uveitis eyes the presence of ROS causing protein and DNA modifications was demonstrated. The increased ROS levels during inflammation could be due to increased oxygen consumption or decreased antioxidative defense in the concerned tissue [4, 5]. Retinopathy of prematurity affects premature infants. Pathological angiogenesis is a key feature of this disease [6]. There is considerable evidence that increased ROS production in the retina participates in retinal angiogenesis, although the mechanisms by which this occurs are not fully understood. ROS might be produced by a number of pathways, including the mitochondrial electron transport chain, cytochrome P450, xanthine oxidase, and uncoupled nitric oxide synthase [7]. Dry age-related macular degeneration and exfoliation syndrome are two common and complex age-related conditions that can cause irreversible vision loss. Oxidative stress refers to cellular or molecular damage caused by ROS, which especially occurs in age-related conditions as a result of an imbalance between the production of ROS and the antioxidant defense response [8]. The authors suggested that although the mechanism responsible for production of the abnormal drusen characteristic for age-related macular degeneration still remained elusive, understanding the early molecular events in their pathogenesis and the exact role of oxidative stress might provide novel opportunities for therapeutic intervention in prevention of disease progression. Glaucoma is an important cause for irreversible blindness worldwide. There appears good evidence that reduction in the antioxidant properties of the trabecular meshwork region of the eye correlates to the progression of primary open-angle glaucoma [9]. Trabecular meshwork is exposed to chronic oxidative stress, particularly to exogenous sources of oxidative stress including the presence of high physiological concentrations of hydrogen peroxide in the aqueous humor which may be induced after light exposure of the anterior segment. Thus trabecular meshwork containing important antioxidant enzymes, such as catalase, superoxide dismutase, and glutathione peroxidase, represents an important defence system against oxidative stress [10]. Decreased antioxidant defense and increased oxidative stress system may play an important role in the pathogenesis of primary open-angle glaucoma [11].

To corneal diseases, associated with oxidative stress and in many cases with corneal aging, belong corneal inflammation, dry eye disease, keratoconus, Fuchs' endothelial dystrophy, and bullous keratopathy [12–18]. In acute corneal inflammation ROS are highly involved. Alio et al. [12] employed the antioxidant therapy with superoxide dismutase and dimethylthiourea for the healing of corneal ulcers evoked by sodium hydroxide. Topical antioxidant therapy was effective in reducing the inflammatory corneal reaction. Augustin et al. [13] studied the oxidative reactions in tears of patients with dry eye disease and confirmed a marked increase of inflammatory activity in the tear film of patients suffering from dry eye. Lipid peroxide levels and myeloperoxidase activity, as parameters for oxidative tissue damage and inflammatory activity, were determined. The authors concluded that both oxidative tissue damage and polymorphonuclear leukocytes indicate an oxidative potential appearing in the tear film of patients with dry eyes. These reactions lead to severe damage of the eye. Free radicals and inflammation may be involved in the pathogenesis or in the self-propagation of the dry eye disease [14–16]. Keratoconus is an eye condition in which the cornea progressively thins resulting in visual impairment. According to Arnal et al. [17] lipid peroxidation products and decreased antioxidant capacity found in keratoconus corneas point to the suggestion that oxidative stress might be involved in the development of this disease. Fuchs' corneal endothelial dystrophy is a slowly progressing corneal dystrophy. In corneas numerous guttae may be present on the posterior surface of Descemet's membrane and also cysts in the corneal epithelium. In bullous keratopathy, small vesicles, or bullae, are formed in the cornea with endothelial dysfunction. Buddi et al. [18] studied the role of oxidative stress in these diseases. Bullous keratopathy corneas displayed byproducts of lipid peroxidation but not peroxynitrite. Conversely, Fuchs' dystrophy corneas displayed byproducts of peroxynitrite with little lipid peroxidation. According the authors these data suggest that oxidative damage occurs within each group of diseased corneas; however, each disease exhibits a distinctive profile.

Numerous keratitis evoked by external influences mainly UV radiation from sunlight and chemical injuries often occur in clinical practice and threaten vision. In the healthy cornea a physiological balance between antioxidant and prooxidant mechanisms exists. However, this balance may be disturbed under various corneal injuries or diseases, in which oxidative stress is involved [18–21]. The amount of antioxidants is decreased and the antioxidant/prooxidant imbalance appears in the cornea. Reactive oxygen species present in abundant amount at the ocular surface are insufficiently cleaved contributing to induction of proinflammatory cytokines in the cornea, generation as well as activation of various proteolytic enzymes (metalloproteinases, serine proteases) and nitric oxide synthases, enzymes that generate nitric oxide [17, 19, 20]. Toxic oxygen and nitrogen products with proteolytic enzymes degrade the cornea [18, 21]. Corneal hydration and light absorption are increased [22].

This review is devoted to diseases and injuries of corneas in which oxidative stress plays an important role. The particular attention is turned to changes in corneal optical properties decreasing visual acuity. New approaches in the treatment of corneal oxidative injuries are presented.

## 2. The Role of UVB Irradiation of the Eye in Oxidative Damage of the Cornea, Changes in Corneal Optical Properties, and Drugs Employed for Suppression of UVB-Induced Oxidative Injury

The cornea is directly exposed to UV radiation of longer wavelength (UVA rays, 320–400 nm) as well as shorter wavelength (UVB rays, 295–320 nm). The cornea absorbs approximately 80% of UVB rays and 34% of UVA rays and acts as a UVB filter. The aqueous humor, containing ascorbic acid, proteins, and some amino acids, also absorbs UVB radiation. The other ocular tissues (particularly the lens) absorb 66% of UVA rays and 20% of UVB rays. The lens acts to filter light between 300 and 400 nm (particularly UVA, a small amount of UVB) from reaching the retina [23].

In healthy corneas the processes of absorption and detoxification of UV radiation in the anterior eye segment are ensured by tissue components and fluids, of which both low-molecular weight and also high-molecular weight antioxidants (antioxidant enzymes) are involved. Of anterior eye segment tissue components, the corneal layers (particularly the epithelium) play the key role in the protection of the inner eye against the effect of UVB rays [24–26]. Among low-molecular weight antioxidants, ascorbic acid (present in high amounts in the cornea) is an important scavenger of reactive oxygen species [27–30]. Besides ascorbic acid, DL-alpha-tocopherol acetate (vitamin E) also protects against the harmful effects of ROS. Vitamin E is a free radical scavenger and protects cells by breaking the chains of reactive oxygen species [31]. Of high-molecular weight antioxidants, superoxide dismutase, catalase, and glutathione peroxidase are the key antioxidant enzymes that protect the cornea against the damage induced by ROS [32]. Superoxide dismutase catalyzes the superoxide radical dismutation to hydrogen peroxide and molecular oxygen. Glutathione peroxidase and catalase are potent scavengers of hydrogen peroxide. Corneal aldehyde dehydrogenase 3A1 has an important role in the detoxification of ultraviolet-induced peroxidic aldehydes and in corneal UVB absorption [33–36].

Under normal conditions these mechanisms present in the cornea protect the inner eye against damaging effect of UV radiation and the oxidative damage induced by them. However, when threshold levels of UV radiation are exceeded, corneal disturbances appear. It is well known from clinical practice as well as from experimental studies that excessive exposure of the eye to UV radiation evokes intracorneal inflammation, known as photokeratitis. This acute corneal response to solar UV radiation reaching the eye is caused by UVB radiation [37]. The initial* in vivo* (clinical) signs of photokeratitis are due to lost or damaged corneal epithelial cells with other signs resulting from this primary response [38]. According to Pitts et al. [39] the threshold radiant exposure of rabbit corneas rises very rapidly from 0.022 J/cm(2) at 300 nm to 10.99 J/cm(2) at 335 nm. Radiant exposures exceeding 2× threshold radiant exposure resulted in irreversible corneal damage. Cullen [38] and Doughty and Cullen [40] describe that the endothelial ultraviolet damage threshold was approximately 0.125 J/cm(2) (at the anterior corneal surface).

**Figure 1:**
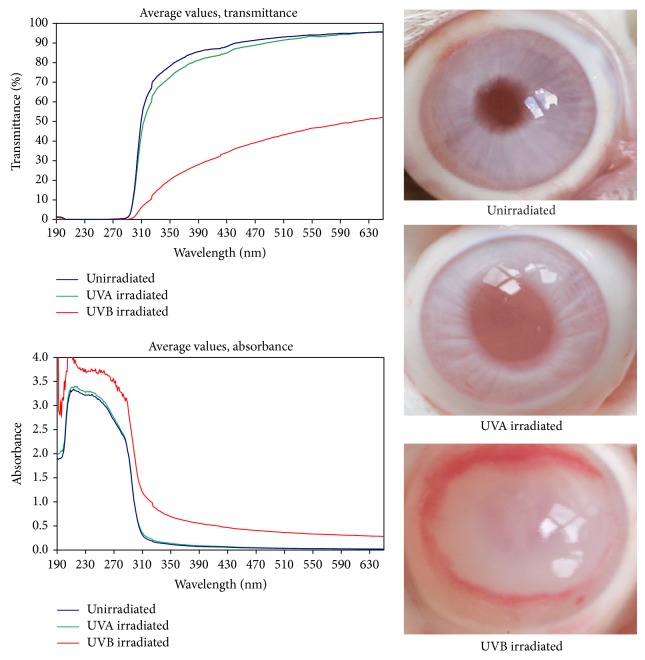
Averaged absorption spectra of rabbit corneas, expressed as the transmittance *T* = *T*(*λ*) or absorbance *A* = *A*(*λ*), after irradiation of the eye with UVB rays (312 nm) or UVA rays (365 nm), 1.0 J/cm(2) once daily for four days. The spectrum of unirradiated corneas (healthy eyes; mean from 12 measurements) is also included for comparison. Note that, for wavelengths shorter than about 300 nm, the spectra show the instrumental stray light error rather than the corneal optical properties (absorption spectra of rabbit corneas were obtained using a spectrophotometrical method described by Čejka et al. [22]). The increased corneal light absorption after UVB irradiation goes in parallel with the changed corneal transparency. The cornea is vascularized at the periphery. In contrast, a similar dose of UVA radiation did not significantly change corneal light absorption or corneal transparency.

In experimental studies with the irradiation of the rabbit eye with UVB radiation or UVA radiation (daily dose of 1.01 J/cm(2) for four days) it was found that UVB radiation evoked the increase in corneal hydration and light absorption and initiated the intracorneal inflammation [22]. In contrast, UVA radiation of similar doses did not statistically significantly change the corneal hydration and light absorption properties from those seen in normal corneas. Similar findings were obtained with twofold higher UVA dose (daily dose of 2.02 J/cm(2) during five days). The thickness of the corneal centers after irradiation with UVA rays (both doses) was not significantly changed compared to normal corneas. The corneas remained transparent and undamaged [41]. In Figure 1 differences between the irradiation of the rabbit eye with UVB and UVA rays are shown.

Čejka et al. [22] studied corneal light absorption after the irradiation of the rabbit cornea with UVB radiation and showed that the protein content in the corneal epithelium was dramatically reduced following UVB irradiation. The drop in protein content of the epithelium may be due to the fewer epithelial cells after 5 days of irradiation.

According to Rogers et al. [42] already subsolar UVB radiation caused irreversible damage to the corneal epithelium. Podskochy et al. [43] described that apoptosis appeared to be a mechanism of corneal cell death after UVB rays. Of other morphological disturbances, Kennedy et al. [44] described that acute UV irradiation exposure results in the induction of cornea-derived proinflammatory cytokines. The local release of proinflammatory cytokines by cells in the irradiated cornea might be responsible for UV-mediated corneal inflammation. Hong et al. [45] found that after epithelial injury proinflammatory chemokines are produced by keratocytes, which probably trigger the influx of inflammatory cells into the corneal stroma. This is in accord with results of Čejková et al. [46]. In the cornea repeatedly irradiated with UVB rays numerous inflammatory cells were present in the corneal stroma, particularly in the anterior region. In UVB-irradiated corneas, morphological disturbances went parallel with enzymatic disorders, particularly in antioxidants. Corneal antioxidants detoxify reactive oxygen species and thus protect the inner eye against the oxidative injury [19]. Repeated exposure of the rabbit corneal cells to UVB rays led to the significant decrease in activities of glutathione peroxidase (an enzyme cleaving hydrogen peroxide) and superoxide dismutase (an enzymatic scavenger of superoxide) [47, 48]. Also the activity of corneal aldehyde dehydrogenase 3A1, an enzyme which has an important role in the detoxification of ultraviolet-induced peroxidic aldehydes and in corneal UVB absorption [33–36], was dramatically reduced in the mice cornea after UVB irradiation [49]. Of nonenzymatic antioxidants, ascorbic acid is an important scavenger of reactive oxygen species. The protective role of ascorbic acid in the oxidative defense of the eye lies in its reducing properties [23]. After repeated UVB exposure, a significant decrease in ascorbic acid in the cornea and aqueous humor was demonstrated [50, 51]. From the above-mentioned studies, it follows that, in the normal cornea, antioxidants, together with tissue components, absorb and detoxify UVB radiation. However, UVB irradiation leads to a pronounced reduction in antioxidants, which might initiate oxidative injury to the internal parts of the eye, particularly the lens.

Of antioxidant agents, Lodovici et al. [48] described that 4-coumaric acid may be useful in protecting the eye from free radical damage from sunlight through its free radical scavenging and antioxidant properties. From antioxidant agents' perspective for therapeutic use in humans, trehalose has been described to be very effective in protection of the rabbit eye irradiated with UVB rays against oxidative stress [52–54]. Trehalose is a nonreducing disaccharide of glucose that is produced and stored in many lower and higher organisms, although not in mammals. When these organisms are exposed to stress, they adapt by synthesizing huge amounts of trehalose, which helps them to retain the cellular integrity [52]. Trehalose treatment of UVB-irradiated eye suppressed proinflammatory cytokine induction in the cornea, contributing to reduced matrix metalloproteinase and xanthine oxidase expression in the UVB-irradiated corneal epithelium and to the decreased development of an antioxidant/prooxidant imbalance [53]. The overexpression of heat shock protein 70 found in UVB-irradiated cornea after buffered saline treatment was reduced after trehalose application [54]. Trehalose also reduced very effectively changes in corneal optical properties caused by increased corneal hydration and light absorption [52]. Of other antioxidant agents, high-molecular weight hyaluronic acid was showed as a potential substance for protecting corneal cells from UVB-induced oxidative injury [55, 56]. Hyaluronic acid provided anti-inflammatory and antiapoptotic signals to cells exposed to UVB rays; however, it had no significant effect on ROS levels [55].

## 3. The Role of Chemical Injuries in the Appearance of Oxidative Stress in the Cornea, Changes in Corneal Optical Properties, and Drugs Employed for the Treatment of Alkali-Induced Oxidative Damage

**Figure 2:**
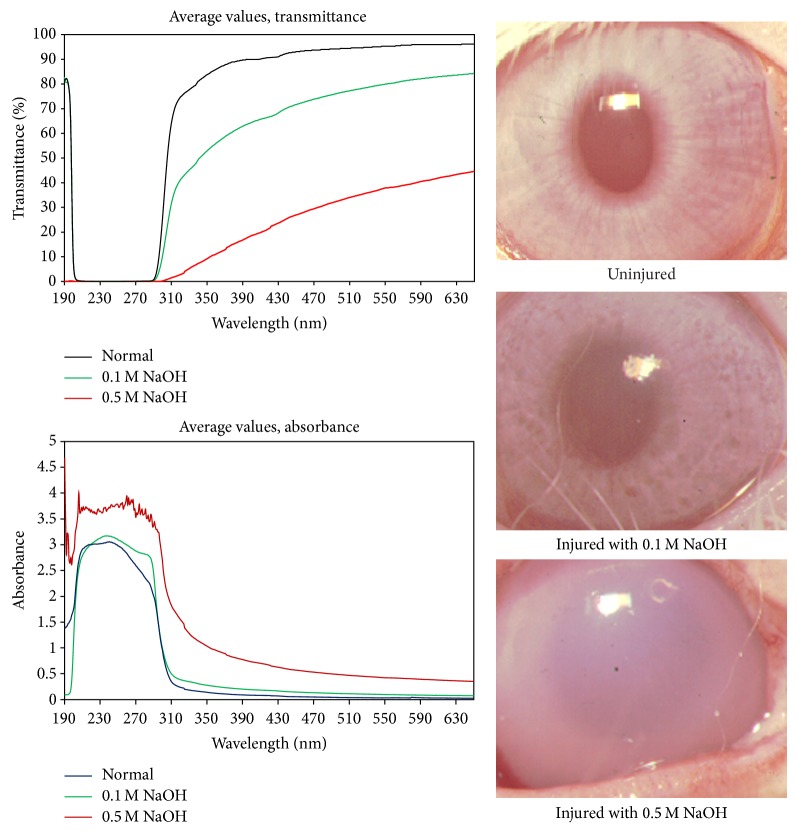
Averaged absorption spectra of rabbit corneas, expressed as the transmittance *T* = *T*(*λ*) or absorbance *A* = *A*(*λ*), after alkali injury of the rabbit eye with 0.1 M or 0.5 M NaOH (10 drops during 1 min and then the eye was rinsed with tap water). The spectrum of unirradiated corneas (healthy eyes; mean from 6 measurements) is also included for comparison. Note that, for wavelengths shorter than about 300 nm, the spectra show the instrumental stray light error rather than the corneal optical properties (absorption spectra of rabbit corneas were obtained using a spectrophotometrical method described by Čejka et al. [22]). The corneal light absorption increased along with the increase in the concentration of the alkali. Also, the changes in corneal transparency proceeded in parallel with increasing concentrations of alkali.

It is generally known from clinical practice that chemical injuries of the cornea (particularly with alkalis) are dangerous to the eye and threaten vision. Čejka et al. [57] described that already low concentrations of alkali changed corneal hydration and corneal light absorption. These authors found that after dropping 0.1 M NaOH onto the surface of the healthy rabbit cornea, increased corneal hydration, as measured by a pachymeter according to the central corneal thickness, appeared 24 h after the first application and the cornea became opaque. The extent of corneal hydration together with changes in corneal light absorption increased along with the daily application of alkali. On the 3rd day of the experiment significantly increased corneal hydration and increased corneal light absorption were found (compared to untreated healthy corneas). In contrast, after the application of the same concentration of diluted acid (0.1 M HCl), these changes, even if apparent, did not reach significance. In Figure 2 is shown the dependence of changes in corneal light absorption and corneal transparency on the severity of corneal alkali injury.

Chemically, alkali dissociates into a hydroxyl ion and cation in the eye. The hydroxyl ion saponifies cell membrane fatty acids, while cation interacts with stromal collagen and glycosaminoglycans. This interaction facilitates deeper penetration of alkali into and through the cornea and into the deeper parts of the eye [58]. According to these authors subsequent hydration of corneal glycosaminoglycans results in stromal haze. Alkali causes collagen fibril distortion and shortening, leading to trabecular meshwork alterations that can result in increased intraocular pressure. The transparency of the cornea is dependent on the level of corneal hydration. During corneal swelling, light scattering increases. Meek et al. [59] describe that although this scattering is ascribed to the disruption caused by the arrangement of the collagen fibrils, light scattering could increase if there is an increased mismatch in the refractive indexes of the collagen fibrils and the material between them. These authors examined refractive index of the healthy bovine cornea versus hydrated bovine cornea. The refractive index of the stroma and its constituent extrafibrillar material reduces as solvent enters the tissue and the overall refractive index of the bovine stroma *n*_*s*_ depends on the hydration H (mg water/mg dry weight) according to following equation: *n*_*s*_ = 1.335 + 0.04/(0.22 + 0.24 H). It was found that when the hydration of the corneal stroma increases from H = 3.2 (hydration of the healthy corneal stroma) to H = 8.0 (the hydration of swollen corneal stroma), the ratio of the refractive index of collagen fibrils to that of the material between them increases from 1.041 to 1.052. The authors [59] concluded that this change makes only a small contribution to the large increase in light scattering observed when the cornea swells to H = 8. Also Kim et al. [60] described variation of corneal refractive index with hydration and the authors considered the measurement of refractive index as a quantitative indicator of corneal hydration. In this connection it is necessary to mention that the refractive index of the cornea is not uniform. The mean refractive index of the epithelium, stromal anterior and posterior surfaces in the human cornea were 1.401 (SD +/− 0.005), 1.380 (SD +/− 0.005), and 1.373 (SD +/− 0.001). The varying refractive index does not significantly affect the total optical power of the cornea [61].

Changes in corneal hydration and light absorption were associated with oxidative stress appearing in the cornea early after the injury with low concentrated alkali [62, 63]. Kubota et al. [62] found enhanced ROS production immediately after an injury with low concentrated alkali in the mouse cornea, as shown by increased dihydroethidium fluorescence indicative of superoxide production and increased levels of nuclear factor kappa-light-chain-enhancer of activated B cells, a protein complex that controls the transcription of DNA. Also, monocyte chemoattractant protein-1 and vascular endothelial growth factor were significantly enhanced, pointing to corneal angiogenesis. Immediate antioxidant therapy of the alkali-injured cornea with H2-enriched irrigation solution facilitated corneal healing [62]. Cejkova et al. [63] demonstrated suppression of oxidative injury in the rabbit cornea evoked by alkali using bone marrow mesenchymal stem cells growing on nanofiber scaffolds and transferred onto the alkali-injured corneal surface. Results of these authors showed that in untreated alkali injured corneas following oxidative disturbances appeared: The expression of malondialdehyde and nitrotyrosine (important markers of lipid peroxidation and oxidative stress) occurred in the corneal epithelium. The expression of aldehyde dehydrogenase 3A1 (an important antioxidant enzyme) decreased in the corneal epithelium, particularly in superficial layers, where apoptotic cell death (detected by active caspase-3) was high. Moreover, the expression of matrix metalloproteinase 9 and proinflammatory cytokines was high and numerous inflammatory cells were present in the vascularized corneal stroma. However, when alkali injured corneas were covered with nanofiber scaffolds with rabbit bone marrow mesenchymal stem cells, aldehyde dehydrogenase 3A1 expression remained high in the epithelium (as in the control cornea) and positive expression of the other immunohistochemical markers employed was very low (matrix metalloproteinases) or absent (nitrotyrosine, malondialdehyde, and proinflammatory cytokines). Corneal neovascularization and the infiltration of the corneal stroma with inflammatory cells were significantly suppressed in the injured corneas treated with MSCs compared to the untreated injured ones. Cejkova et al. [63] further described that the increased central corneal thickness together with corneal opalescence appearing after alkali injury returned to normal levels over the course of ten days only in the injured corneas treated with mesenchymal stem cells on nanofiber scaffolds. The expression of genes for the proinflammatory cytokines corresponded to their immunohistochemical findings. The authors [63] concluded that mesenchymal stem cells on nanofiber scaffolds protected the formation of toxic peroxynitrite, lowered apoptotic cell death, and decreased matrix metalloproteinase and proinflammatory cytokine production. This resulted in reduced corneal inflammation as well as neovascularization. Corneas healed with the restoration of transparency and renewal of visual acuity. MSCs proved to be very perspective for the therapeutic use of oxidative corneal injuries evoked by alkali burns. Moreover it was found that mesenchymal stem cells pretreated with interferon-*γ* and administered systematically (intravenously) migrated to the alkali-injured eye and effectively inhibited the acute phase of intracorneal inflammation [64]. Good inhibition of early phase of corneal inflammation after corneal alkali burns was also obtained with subconjunctivally administered mesenchymal stem cells [65].

Further effective antioxidant therapy of alkali-injured corneas was described by Brignole et al. [66] using regenerating agent (RTGA). RGTA enhanced corneal reepithelialization and corneal transparency and* in vitro* RGTA protected conjunctival cells from oxidative stress evoked by benzalkonium chloride by reducing hydrogenium peroxide production and glutathione uptake. Regenerating agents (RGTA) are biopolymers engineered to replace heparan sulfates, obtained by the chemical substitution of dextran, and have been shown to modulate collagen synthesis in several cell-culture and tissue-explant models. RGTA is specifically bound to matrix proteins and growth factors that are destroyed after an injury [67]. Cejkova et al. [68] employed RGTA (CACICOL20) for the healing of the rabbit cornea injured with lower as well as higher concentrations of alkali (NaOH). These authors showed that, compared to control (unaffected) corneas, following the application of low alkali concentration, the expression of urokinase-type plasminogen activator, metalloproteinase 9, nitric oxide synthase, and xanthine oxidase was increased in the injured corneal epithelium of placebo-treated eyes, whereas the expression of antioxidant enzymes was reduced. Nitrotyrosine and malondialdehyde stainings appeared in the corneal epithelium. RGTA application suppressed the antioxidant/prooxidant imbalance and reduced the expression of the above-mentioned immunohistochemical markers. It was further described [68] that corneal thickness increased after alkali injury and decreased during RGTA application. Following the injury with the high alkali concentration, corneal inflammation and neovascularization were highly pronounced in placebo-treated corneas, whereas in RGTA-treated corneas they were significantly suppressed. When RGTA or placebo application was started later after alkali injury and corneas were ulcerated, subsequent RGTA treatment healed the majority of them. RGTA facilitated the healing of injured corneas via a reduction of proteolytic, oxidative, and nitrosative damage [68]. Very similar results with RGTA in the healing of corneal oxidative injuries were described by Brignole-Baudouin et al. [69]. These authors found that RGTA decreased corneal inflammation, accelerated corneal healing, and enhanced corneal reepithelialization. RGTA was very well tolerated; no signs of ocular irritations were observed. RGTA was employed for the treatment of neurotrophic keratopathy, a degenerative disease of the corneal epithelium in humans. RGTA seemed to be a potentially useful, alternative, noninvasive therapeutic approach for the treatment of this disease [70].

Previous papers recommended citrate or ascorbate/citrate treatment of oxidative injuries after severe corneal alkali burns [71–73]. Topical citrate reduced the inflammatory response in the cornea by inhibiting polymorphonuclear leukocytes. Topical ascorbate elevated the depressed level of this vitamin in the alkali-injured cornea. Combination of both drugs suppressed the incidence of corneal ulcerative processes. Alio et al. [12] employed for topical treatment of alkali-injured corneas 0.5% dimethylthiourea and 0.2% superoxide dismutase. Dimethylthiourea was shown to be effective in suppression of corneal inflammation and superoxide dismutase in the reduction of corneal ulcers.

Very good experiences in oxidative disorder management after corneal alkali burns have been achieved with tear supplements, particularly based on hyaluronic acid, topically administered corticosteroids, and prophylactic antibiotic use [74–84]. Chemically modified and cross-linked hyaluronan gel improved corneal wound healing after alkali injuries in rabbits [74]. The wound closure rate and thickness of the corneal epithelium in eyes treated with hyaluronan gel were significantly greater than in untreated injured eyes. The authors concluded that derivate of hyaluronan could be useful for treating noninfectious corneal injuries. Chung et al. [75, 76] described significantly suppressed stromal inflammation in the alkali-injured cornea with topically used sodium hyaluronate. Both 1% and 2% sodium hyaluronate had a statistically significant positive influence on the epithelial resurfacing, especially during the late healing phase. Stromal healing was determined by counting polymorphonuclear leukocytes and keratocytes in the central and marginal wound areas. In stromal healing, 1% sodium hyaluronate suppressed the stromal polymorphonuclear infiltration and enhanced the keratocyte repopulation during corneal alkali wound healing. Sekundo et al. [77] examined the effect of antioxidants (allopurinol and acetylcysteine) and corticosteroids (prednisolone acetate) in eye drops in the healing of corneal alkali burns. These drugs were effective particularly in the early treatment of experimental corneal burns. All three substances significantly reduced the number of histologically visible inflammatory cells compared to control group. Saud et al. [78] described a favorable effect of subconjunctival injection of a long-acting synthetic corticosteroid triamcinolone after corneal alkali burns in rabbits. Triamcinolone was very effective for the treatment of acute ocular burn because it reduced the corneal inflammatory process, opacity, and vascularization. Of antibiotics, oral treatment with doxycycline, belonging to tetracycline antibiotic class, was found to be very effective in preventing corneal angiogenesis and inflammation of alkali-burned corneas [79, 80]. Dan et al. [79] described in rats the efficacy of oral doxycycline treatment as compared to oral and topical dexamethasone treatment in inhibiting corneal neovascularization following corneal alkali burn. The inhibition of neovascularization was greater in both dexamethasone groups compared to doxycycline group. However, epithelial healing was significantly more rapid in the doxycycline group compared to both dexamethasone groups. Epithelial ulceration was apparent in both oral and topically treated dexamethasone rats, but not in doxycycline-treated rats. Ling et al. [80] explored the inhibitory effects of doxycycline on allograft rejection in alkali-burned cornea beds. Doxycycline had a significant role in preventing corneal angiogenesis and inflammation in alkali-burned corneal beds, which resulted in higher allograft survival rates. Su et al. [81] described that doxycycline enhanced the inhibitory effect of bevacizumab (an angiogenesis inhibitor) on corneal neovascularization and expression of polymorphonuclear leukocytes in the burned corneal stroma. Combination therapy showed better inhibitory effects and accelerated corneal healing. Shin et al. [82] investigated the protective effect of rapamycin (a macrolide with immunosuppressant function) against alkali-induced corneal damage in mice. Rapamycin reduced proinflammatory cytokines in the cornea and decreased corneal fibrosis. Corneal neovascularization and corneal opacity were suppressed. Shahriari et al. [83] compared corneal epithelial wound closure rates in rabbit eyes after application of amniotic membrane suspension, autologous serum, or preservative-free artificial tears. These studies showed that alkali-injured corneal epithelial wounds healed faster when treated with amniotic membrane suspension than with autologous serum or preservative-free artificial tears. Oh et al. [84] evaluated the effect of umbilical cord serum eye drops on corneal healing and haze in mouse model of ocular chemical burn and compared these eye drops with peripheral blood serum eye drops or artificial tears. Umbilical cord serum eye drops were more effective in improving corneal wound healing and reducing corneal haze compared with peripheral blood serum eye drops or artificial tears in experimental chemical burns.

## 4. Concluding Remarks

Oxidative stress in the cornea appearing after the influence of various environmental noxae may be involved in the initiation as well as propagation of corneal disturbances accompanied by changes in corneal optical properties and decrease in visual acuity or even vision loss. The drugs with antioxidant effects suppressing the oxidative injury are necessary for corneal healing and renewal of corneal optical properties inevitable for vision restoration.
